# Anatomy and morphology of iliolumbar ligament

**DOI:** 10.1007/s00276-022-03070-y

**Published:** 2023-01-02

**Authors:** K. Dąbrowski, B. Ciszek

**Affiliations:** 1 Department of Descriptive and Clinical Anatomy, Center for Biostructure Research, Medical University of Warsaw, Warsaw, Poland; 2 Department of Neurosurgery in Bogdanowicz Children’s Hospital, Warsaw, Poland

**Keywords:** Iliolumbar, Ligament, Morphology, Variability, Anatomy, Pelvis

## Abstract

**Purpose:**

To address limited amount of available data and contradictory statements in published works 60 Iliolumbar ligaments extracted from 30 cadavers were examined to describe their insertions and morphology.

**Methods:**

The ligaments were removed during the standard autopsy procedures with a use of an oscillating saw, a chisel and a scalpel. The specimens were photographed before the extraction and measured alongside their anterior margin. Next, they were preserved in formaldehyde, stripped of other soft tissues and then examined, photographed and described.

**Results:**

The mean length of the ligaments was 31.7 mm. 44 specimens were described as single-banded, 13 as double-banded and 3 as other. In 24 cases costal process of L_V_ has been fixed to the iliac plate by short ligamentous bands. In 38 cases there was a thick fibrous membrane connected to the ligament. No legitimate insertions on L_IV_ vertebra were observed.

**Conclusions:**

Typical iliolumbar ligament consists of a single ligamentous band. Most common variability of the ligament consist of two bands. In approximately 40% of cases the costal process of L_V_ can be additionally stabilized to the iliac plate by short, strong ligamentous bands. In 63% of cases a connection between the iliolumbar ligament and a fibrous membrane placed in the frontal plane, superiorly to the ligament, has been observed. There seems to be no convincing proof of existence of the insertion of the iliolumbar ligament on the L_IV_ vertebra.

## Introduction

The iliolumbar ligament is commonly described as a ligamentous band connecting fourth and fifth lumbar vertebrae with the ilium on the same side. Despite being possibly one of the most important ligaments in human body—being the evolutionarily crucial structure allowing erect posture and bipedal movement [7, 10, 12, 13, 17]—the research on it seems to be scarce and results often turn out to be contradictory.

Most authors describe the proximal insertion as being around the anterior side of the costal process of L_V_ vertebra, be it anterior–superior, anterior–lateral or anterior–inferior–lateral side [4, 6, 11]. From there the ligament supposedly travels toward its insertions on the iliac crest in form of, depending on the author, one, two, three, five or even seven bands [4, 7, 11, 12, 14–18].

Interestingly, although most authors repeat the description of the ligament having its proximal insertions on the costal process of both fifth lumbar (L_V_) and fourth lumbar (L_IV_) vertebrae, most of them notes that they cannot confirm such occurrence. During their research they either were unable to find a single case of such ligament, found singular cases, making it a rare variability, or have observed that any connections between the iliolumbar ligament and the L_IV_ vertebra are weak, questionable in nature and should be omitted [3, 7, 9, 14, 15].

This study aims to gather modern data and compare the results to the existing observations of previous authors in hope of helping to solve the aforementioned discrepancies.

## Materials and methods

The iliolumbar ligaments have been extracted from 20 male and 10 female cadavers between 17 and 70 years of age. The mean age was 50.9 years with Standard Deviation (SD) of 12.38. The mean height was 171.4 cm with SD of 10.02 cm. Overall, 60 ligaments were examined.

**Fig. 1:**
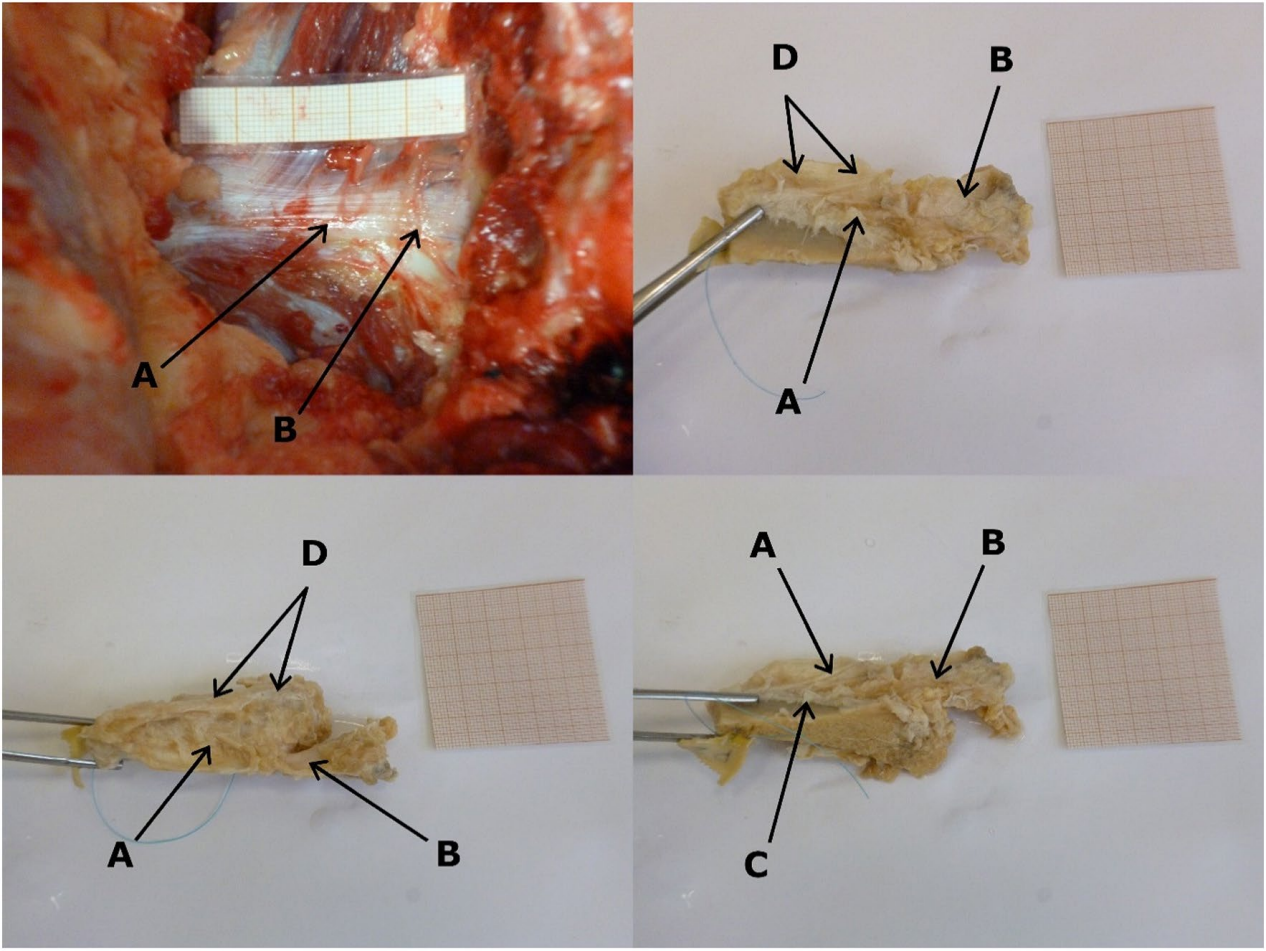
Example of examination and findings. **A** Singular, rectangular band. **B** Insertion at the apex of costal process of L_V_. **C** Insertion at the iliac crest. **D** Fibrous membrane at the superior margin of the ligament

The extraction has been performed during standard autopsy at the Department of Forensic Medicine. After removal of the internal organs the iliolumbar ligaments and their insertions were uncovered with a scalpel and the measurements and photographs were taken. Afterward, the ligaments with their insertions were separated from the rest of the body by an oscillating saw, a scalpel and a chisel. Extracted material has been preserved in formaldehyde and manually stripped of soft tissues other than the ligament. Prepared ligaments were photographed and their morphology described (as shown in Fig. 1).

## Results

Mean length of the iliolumbar ligament measured from the tip of the costal process to the furthest point of insertion on the iliac plate was 31.7 mm with SD of 9 mm. By sex, mean length was 31.4 mm with 10.6 mm SD for males and 31.9 mm with 5.4 mm SD for females. Fisher’s test results for the sexual dimorphism in length of the ligament was: *p* value = 0.039, which, assuming the border of statistical significance being *p* value = 0.05, proves to be significant. However, there were no significant differences between the left and the right ligaments.

44 specimens (73.3%) were described as single-banded, out of which 28 (46.6%) presented either visible or palpable oval thickening at the anterior margin of the ligament. 30 specimens in the single-banded group were described as rectangle-shaped, 13 as fan-shaped and one as mixed between the two types.

13 specimens (21.6%) were recognized as double-banded with majority having oval-shaped anterior band and either rectangle-shaped (4 specimens) or fan-shaped (5 specimens) posterior band. Other combinations could be described as miscellaneous.

Remaining specimens (5%) were described as multi-banded, and mostly fan-shaped.

**Fig. 2:**
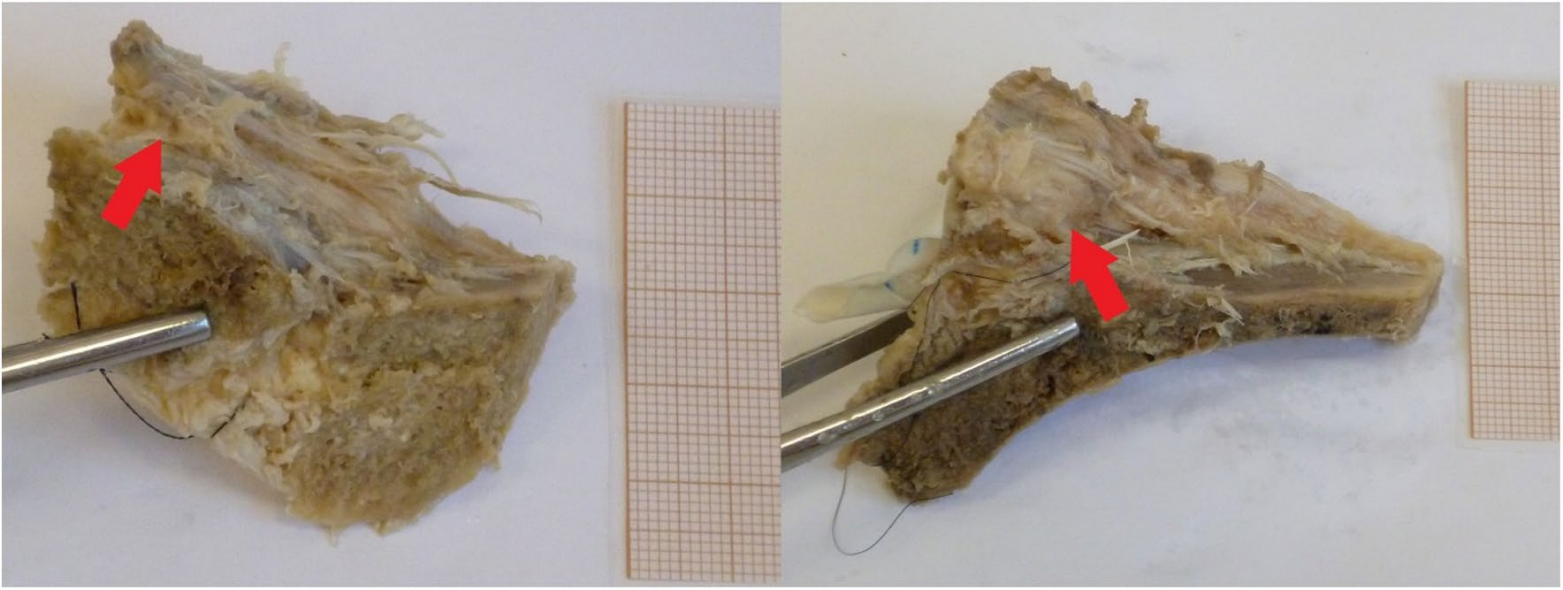
Examples of the short collagenous bands connecting the costal process with the surface of the iliac crest. Both examples are left ligaments, shown as if observed from the caudal direction

In 24 ligaments (40%) multiple short collagenous bands were observed fixating the costal process to the surface of the iliac plate (Fig. 2)

**Fig. 3:**
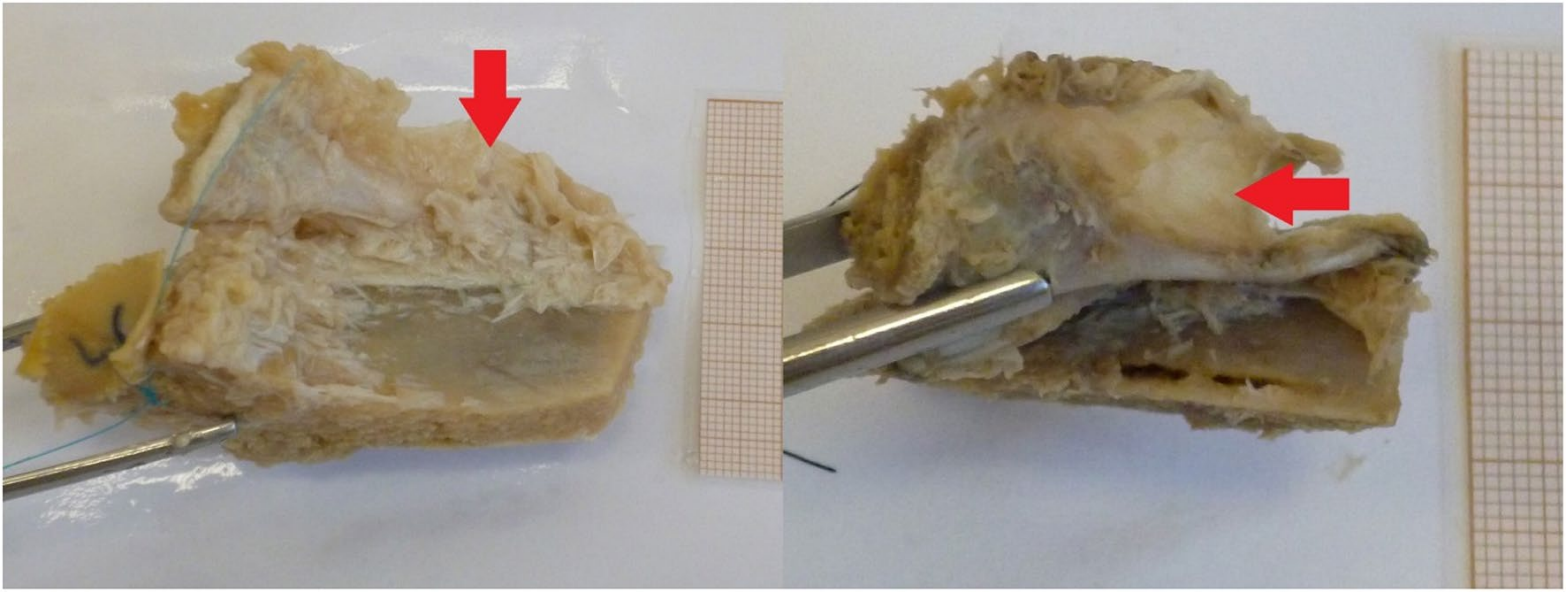
Examples of the membrane with different levels of adipose saturation. Both examples are left ligaments, shown as if the cadaver was lying on its back

In 38 cases (63%) a membranous connection between the ligament and the L_IV_ vertebra has been observed, yet none could have been considered a legitimate insertion, following Aihara’s [1–3] opinion, not having biomechanically relevant amount of collagenous bands anchored onto the periosteum.

## Discussion

When it comes to comparison of the results of this research with other published papers, there are certain difficulties. The main one stems from the fact that most measurements published by other authors were taken on two-banded ligaments, differentiating between the anterior and posterior band. Length of such ligaments seems to fall between 10 and 15 mm [6], 29–30 mm [7] and 30–40 mm [8, 14] of length for the anterior band and either 15–20 mm [6] or 25–26 mm of length [7] for the posterior band. In this research the measurement was taken from the apex of the L_V_ costal process to the furthest point of insertion on the iliac crest along the anterior margin of the structure. It was considered the most reliable and universal measurement that allows relating the results to those of the other authors regardless of the type of the ligament in question.

The length of the measured ligaments had a mean of 31.7 mm on the left (SD of 9 mm) and 32.3 mm on the right (SD of 9.5 mm) which results in the overall mean of 32 mm—being comparable to Hammer’s results. Considering the standard deviation it is hard to consider the results to be matching the range set by the other authors.

Most closely, however, the results of this research seem to coincide with those of Kiter from 2010. Kiter in his paper describes the mean length of the anterior band as 29.86 m and the lean length of the posterior band as 23.71 mm in two-banded ligaments, and the mean length of a single-banded ligament as 32.13 mm [10]. These results fall closely to those of Hammer when it comes to two-banded ligaments and to results described in this paper when it comes to single-banded ligaments.

Regarding the general structure of the ligament, there seems to be a big dissonance between the publishing authors regarding the amount of the fibrous bands included within. Bogduk, Twomey, Vora and Yamamoto describe five bands—superior, inferior, anterior, posterior and transverse [16–18]. However, more popular nowadays seems to be a description of two bands—a wide and flattened anterior band and an oval posterior band [4, 7, 11, 14, 15]. In this examination the most common variability turned out to be a single, rectangle-shaped band with parallel fibers and similar area of insertion on both ends. Such variability occurred in 50% (30 out of 60 specimen). Moreover, in 13 more cases a single fan-shaped band, with area of iliac insertion many times bigger than the vertebral insertion and non-parallel fibers, have been found, which amounts to 21.6%. One case has been described as a mix between the two mentioned types.

It means that 44 out of 60 specimens (73.3%) consisted of a single band. This observation provides different information from vast majority of papers and could be considered an outlier if not for the fact that there are other authors who had similar results. Basadonna, Fujiwara and Kiter, despite their inclination toward the two-band model, have all observed a single band as most common in their material [4, 6, 10, 16].

Out of the rest of the specimens, 13 (21.7%) have been recognized as two-band ligaments, and 3 (5%) as multiple-band ligaments. Out of the mentioned 13 two-bands, 9 had their anterior band be oval-shaped and posterior band be either rectangle-shaped (4 specimen) or fan-shaped (5 specimen). This seems to be the exact opposite of the typical description of the two-band model which matched only two of the examined ligaments, both belonging to a 70-year-old female, making it a possible outlier.

The main issue with such classification seems to be its subjectivity. What one observer can describe as a rectangle-shaped band with a slight spreading of the fibers, another can classify as an obvious fan-shaped specimen. Similarly, since no rectangle-shaped band has four actual right angles, differentiation between a rectangle-shaped and an oval-shaped band provides a dilemma which needs to be settled by the author. The same situation applies to the amount of the bands. In this paper the small perforations within the ligaments or singular ligamentous fibers have not been considered to constitute separate bands, following opinions of other authors that most of these could not possibly affect the biomechanics of the ligament [1, 2, 5, 6].

During the procedures of this examination another interesting observation has been made. In 38 out of 60 specimen (63.3%) there was a fibrous membrane attached to either the upper margin of the ligament or to its insertion on the iliac crest, and spreading upward toward the costal processes of L_V_ and L_IV_ and the intertransverse ligaments connecting them (Fig. 3).

In most cases the membrane was thick, white-yellowish in color and strongly laced with adipose tissue, although in some cases, it was more akin to a particularly strong fascia.

Assuming that the amount of adipose tissue is simply related to the age and lifestyle of the examined individuals, it is possible that aforementioned membrane was a connection between the iliolumbar ligament and the fascia of quadratus lumborum muscle that had been observed before [6, 7]. Vleeming describes a fibrous connection between the ligament, the lumbar vertebrae and the capsule of sacroiliac joint as a commonly appearing structure [16] which could possibly match the current observation, especially since the possibility that in some cases the membrane remained unnoticed and has been damaged or removed during this research cannot be excluded.

Despite that it needs to be stated that during this research no legitimate insertion of the iliolumbar ligament on the L_IV_ vertebra has been observed. This supposed point of insertion has been described in multiple textbooks and mentioned by many authors, yet upon closer inspection of the bibliography on the subject many authors admit either to not being able to find a single case in their material [7, 14] or finding very few cases of questionable quality [3, 9, 15]. The most favourable results belong to Pool-Goudzwaard who claims to have found the L_IV_ insertions in about 30% of her material. Yet in the same paper she describes observing ligamentous connections between the iliolumbar ligament and the L_IV_–L_V_ intertransverse ligament [12] which creates a possibility that the insertions she observed were in fact parts of the intertransverse ligament as well.

As for the results of this research—the previously described membrane that has been observed to connect the ligament and the L_IV_ for the most part did not include ligamentous fibres in its structure, and in few cases in which they were present, they were singular, thin strands that, following Aihara’s opinion, were too weak and insignificant to have any impact on the biomechanics of the ligament and thus should be considered artifacts rather than insertions [3].

## Conclusion

According to this examination a typical iliolumbar ligament consists of a single ligamentous band connecting the apex of the costal process of L_V_ to the iliac crest, with a length of 31.7 mm in men and 31.9 mm in women, which is a small but significant difference between sexes.

Most common variability of the ligament consist of two bands, with anterior band being oval-shaped and posterior band being either rectangle-shaped or fan-shaped.

In many cases the costal process of L_V_ has been additionally stabilized to the iliac plate by short, strong ligamentous bands placed along the sagittal axis.

A common connection between the iliolumbar ligament and a fibrous membrane placed in the frontal plane, superiorly to the ligament, has been observed.

There seems to be no convincing proof of existence of the insertion of the iliolumbar ligament on the L_IV_ vertebra.

## Data Availability

Data is available for reviewing upon request.
